# Current Aspects in the Pathophysiology and Treatment of Chronic Wounds in Diabetes Mellitus

**DOI:** 10.1155/2013/385641

**Published:** 2013-04-07

**Authors:** Elena Tsourdi, Andreas Barthel, Hannes Rietzsch, Andreas Reichel, Stefan R. Bornstein

**Affiliations:** ^1^Division of Endocrinology, Diabetes, and Bone Diseases, Department of Medicine III, Technical University Medical Center, 01307 Dresden, Germany; ^2^Endokrinologikum Ruhr, 44866 Bochum, Germany

## Abstract

Impaired wound healing is a frequent and very severe problem in patients with diabetes mellitus, yet little is known about the underlying pathomechanisms. In this paper we review the biology of wound healing with particular attention to the pathophysiology of chronic wounds in diabetic patients. The standard treatment of diabetic ulcers includes measures to optimize glycemic control as well as extensive debridement, infection elimination by antibiotic therapy based on wound pathogen cultures, the use of moisture dressings, and offloading high pressure from the wound bed. In this paper we discuss novel adjuvant therapies with particular reference to the use of autologous skin transplants for the treatment of diabetic foot ulcers which do not respond to standard care.

## 1. Introduction

The diabetic foot syndrome is a very severe and common complication in patients with diabetes mellitus with a cumulative lifetime incidence of up to 25 percent [1]. The escalating high rates of diabetes in many parts of the world make diabetic foot ulcers a major and increasing public-health problem. Foot ulcers cause substantial morbidity, impair quality of life, are the most important risk factor for lower-extremity amputation, and result in high treatment costs and enormous economic losses [2]. The factors that delay wound healing in diabetes are multiple and relate both to the impaired glucose metabolism and to the effect of neurovascular complications. Diabetic foot ulcers readily become chronic; all too often these wounds do not heal primarily. Treatment of chronic wounds should be essentially directed against the main etiologic factors responsible for the wound. Management is based on the simple principles of eliminating infection, the use of dressings to maintain a moist wound bed and to absorb exsudate, offloading high pressure from the wound bed, and debridement to accelerate endogenous healing and facilitate the effectiveness of topically applied substances [3]. Nevertheless, there are often cases of persistent diabetic foot ulcers that do not respond to standard care. In such patients, skin replacement therapies either by autologous skin transplantation or by tissue-engineered human skin equivalents are second-line options which could prevent an amputation and should therefore be considered.

## 2. Physiological Process of Wound Healing

The physiological process of wound healing is traditionally divided into four phases: haemostasis, inflammation, proliferation, and maturation or remodelling. These phases are orchestrated by a subtle interplay of cellular and humoral factors [4]. Haemostasis occurs within an hour after injury and is characterized by vasoconstriction and clotting. Platelets not only initiate the clotting cascade but also secrete growth factors and cytokines which initiate healing. The subsequent inflammation phase takes up to seven days and is mediated through neutrophil granulocytes which prevent bacterial contamination and cleanse the wound from cell debris. Monocytes are attracted to the wound by chemotactic factors and differentiate into wound macrophages. The latter not only remove bacteria and nonviable tissue by phagocytosis but also release various growth factors required to stimulate fibroplasia and angiogenesis, thereby providing the basis for the formation of the provisional extracellular matrix (ECM). The proliferation phase is initiated at day 2 after injury and takes up to 20 days. This phase is primarily characterized by tissue granulation and formation of new blood vessels (angiogenesis). The angiogenic process involves growth factors such as platelet-derived growth factor (PDGF), macrophage angiogenesis factor, and angiotensin. Concomitant epithelialisation is then initiated to cover the granulation tissue with a cellular barrier. The last phase involving extensive tissue remodelling lasts from one week to six months after injury. During that phase the provisional wound matrix is replaced with proteoglycan and collagen molecules which readily become organised into thicker bundles resulting in stronger but more rigid scar tissue.

## 3. Pathophysiology of Wound Healing in Diabetes

Wound healing in diabetes is impaired by factors that are both extrinsic and intrinsic to the wound and its biology. Extrinsic factors include repeated trauma or mechanical stress applied to a foot that has been rendered insensitive due to neuropathy as well as ischemia as a result of macro- or microvascular disease [5]. Thickening of the basement membrane of the capillaries and arterioles frequently occurs in individuals with diabetes, resulting in an impaired wound healing and persistent ulcer formation [6]. An important role has been attributed to factors intrinsic to the biology of the chronic wound in diabetes. It has been postulated that hyperglycaemia itself has a deleterious effect on wound healing through the formation of advanced glycation end-products (AGEs) which induce the production of inflammatory molecules (TNF-*α*, IL-1) and interfere with collagen synthesis [7]. Furthermore, Spravchikov et al. showed that exposure to high glucose is associated with changes in cellular morphology, decreased proliferation, and abnormal differentiation of keratinocytes [8], thus revealing another mechanism by which hyperglycaemia may affect wound healing in diabetes. Interestingly, the healing times of leg and foot ulcers are decreased in diabetic patients with lower HbA1c, thereby emphasizing the clinical correlation between hyperglycaemia and impaired wound healing [9]. An altered immune function may also contribute to poor wound healing in patients with diabetes. Decreased chemotaxis, phagocytosis, bacterial killing [10], and reduced heat shock protein expression [11] have been implicated in the early phase of wound healing in diabetes. Fahey et al. demonstrated that altered leukocyte infiltration and wound fluid IL-6 characterize the late inflammatory phases of wound healing in diabetes [12]. It therefore seems that an altered pattern of cytokine appearance in the wound milieu may contribute to delayed wound healing in diabetes. This is substantiated by the fact that altered bioavailability of cytokines and growth factors have been implicated in the pathogenesis of chronic wounds. These signalling molecules are secreted by various cell types to control cellular proliferation, differentiation, migration, and metabolism. Abnormal expression of growth factors has been observed in diabetic foot ulcers [13]. It has been postulated that trapping of growth factors and cytokines by certain macromolecules such as albumin, fibrinogen, and *β*2-macroglobulin may disrupt the healing process [14]. Furthermore, increased degradation of growth factors in wound fluid of diabetic subjects has been discussed as a factor contributing to an impaired wound healing process. For example, Duckworth et al. have reported an increased activity of insulin degrading enzyme (IDE) activity in wound fluid from patients with diabetic foot ulcers [15]. Interestingly, insulin degrading activity in the wound fluid was found to be positively correlated with HbA1c levels, thereby supporting the fact that glucose control is an essential prerequisite for wound healing. In addition, normal wound healing requires a balance between the accumulation of collagenous and noncollagenous extracellular matrix components. Their remodelling is determined by matrix metalloproteinases (MMPs) and the tissue inhibitors of metalloproteinases (TIMPs) [16]. MMPs play essential roles in initial wound debridement as well as in angiogenesis, epithelialization, and remodelling of scar tissue [17]. Several studies reported elevated levels of MMPs and reduced levels of TIMPs in chronic wounds [18] with a similar pattern in wounds of patients with diabetes mellitus [19]. Last but not least, there is also increasing evidence that the resident cells of chronic wounds may undergo phenotypic changes that impair their capacity for proliferation and movement. For example, it has been reported that fibroblasts from venous and pressure ulcers are senescent and have a diminished ability to proliferate with the proliferative capacity being directly correlated to the failure to heal [20]. 

## 4. Standard Treatment Methods in Diabetic Foot Ulcers

The standard treatment of diabetic ulcers includes measures to assess vascular status and optimize glycemic control as well as extensive debridement, infection elimination by antibiotic therapy based on wound pathogen cultures, the use of moisture dressings, and offloading high pressure from the wound bed. Vascular assessment should include palpation of all lower-extremity pulses, including femoral, popliteal, posterior tibial, and dorsalis pedis pulses. A surrogative and more accurate method of diagnosing vascular insufficiency in the lower limbs is the use of the ankle branchial pressure index (ABPI), the results of which can be validated through Doppler waveform and pulse oximetry. In case of significant peripheral arterial disease, therapeutic revascularisation should be undertaken, since adequate vascular supply is essential for wound healing. The correlation between normoglycaemia and facilitated wound healing in diabetes has been discussed in the previous section. The pivotal role of surgical debridement in healing of diabetic foot ulcers is widely acknowledged [21]. The rationale lies in removing necrotic, devitalized wound bed and wound edge tissue that inhibits healing, so that secondary wound healing can be achieved [22]. The determination of organisms responsible for a diabetic foot infection via culture of appropriately collected tissue specimens enables clinicians to make optimal antibiotic choices based on culture and sensitivity results [23]. A recent meta-analysis of randomized controlled trials (RCTs) comparing the effects of different types of wound dressings in the treatment of diabetic foot ulcers found no significant differences between them so that aspects such as the dressing cost and the wound properties should be considered when making a decision [24]. A strong association between the efficacy to offload the foot and clinical outcome is supported through evidence-based guidelines [25].

## 5. Additional Current Treatment Methods in Persistent Diabetic Foot Ulcers

### 5.1. Autologous Skin Transplantation in Diabetic Foot Ulcers

Flaps and grafts are the two principal surgical procedures for skin tissue replacement. A flap is a full-thickness portion of skin sectioned and isolated peripherally and in depth from the surrounding skin, except along one side, called the peduncle. A graft is a section of skin of variable thicknesses and sizes completely detached from its original site and used to cover the zone to be repaired. Particular attention should be paid to mesh grafts which are obtained by passing a whole dermoepidermal explant through a special surgical tool (mesher), thereby increasing the initial surface area of the explanted skin [26]. Skin grafts are traditionally used in the treatment of severe burns. However, a number of studies have recently reported successful managing of large tissue defects in patients with diabetic foot ulcers with microsurgical grafts [27–29]. The process of graft adoption is defined as the adhesion of the graft skin to the recipient wound area and its subsequent vascularization. This process is identical to that of wound healing. Following an initial rejection phase after the skin grafting procedure with massive inflammation, revascularization of the graft starts after 24 to 48 hours. Initially the graft is pale and white but subsequently adopts a pinkish colour which indicates successful adoption in association with firm attachment to the bed. Apart from immune compatibility, basic conditions for graft taking encompass the ability for neoangiogenesis, good adherence of the graft to recipient areas, and hence accurate immobilization of the graft. A graft can only be placed to vital exposed dermis capable of producing granulation tissue. The recipient area must not be infected or excessively exudative. In addition well-functioning haemostasis is required. In fact, any accumulation of exudate or blood underneath the graft jeopardizes its survival as it impedes adherence and penetration of new capillaries. The consequent handling of the transplant is of utter importance. In the first weeks after transplantation, complete removal of pressure is essential. Protective footwear with dully formed inserts can secure adequate offloading of the area of high pressure and protect the transplant.

### 5.2. Tissue-Engineered Human Skin Equivalents in Diabetic Foot Ulcers

In the recent years much attention has been paid to the use of tissue-engineered human skin equivalents in the treatment of diabetic foot ulcers. The first engineered skin substitutes were matrix-based products consisting of cross-linked collagen and glycosaminoglycans. The matrix eventually undergoes degradation, while simultaneously the host's cells invade and proliferate within it. Integra, a product of this category, has shown promising results in deep wounds [30]. The second generation of tissue-engineered skin equivalents consisted of cell-based products, mostly keratinocytes. Marston et al. demonstrated that dermagraft, a cryopreserved human fibroblast-derived dermal substitute, is a safe and effective treatment for diabetic foot ulcers [31]. Veves et al. showed that the application of graft skin (Apligraf)—a human skin equivalent manufactured from cultured living dermis and sequentially cultured epidermis of neonatal foreskins—results in significantly improved healing compared to other available treatments. Moreover, there were no significant side effects [32]. Nevertheless, both products are ultimately rejected, so that their primary task appears to be a transient restoration of the dermis until the patients' keratinocytes can migrate and close the wound.

### 5.3. Bone Marrow-Derived Cells

Another very promising therapeutic option involves the use of bone marrow-derived cells, and recent evidence indicates that bone marrow contains stem cells with the potential for differentiation into a variety of tissues. For example, patients with diabetes are known to have an impaired mobilization of endothelial progenitor cells (EPCs) in the bone marrow and decreased accumulation of these cells in wounds [33, 34]. Bone marrow-derived cells may thus be a valuable and unlimited source of progenitor and/or stem cells [35]. For example, Badiavas and Falanga described that the local application of autologous bone marrow-derived cells resulted in complete wound closure in 3 patients unresponsive to standard therapies including bioengineered skin application and autologous skin grafting [36].

Furthermore, it is assumed that hyperbaric oxygen results in EPC recruitment but does not improve migration of EPC to the wound site. However, in a murine model of diabetes coadministration of stromal cell-derived factor-1-alpha (SDF-1*α*) resulted in homing of the activated EPCs to the wound site [37]. These data suggest that combining oxygen therapy with SDF-1*α* may improve wound healing in patients with diabetes. 

Another novel interesting approach consists of lineage commitment of stem cells to the keratinocyte lineage. This can be achieved through exposure of the stem cells to a mixture of cytokines, growth factors, and extracellular matrix components in vitro and has been attempted with only moderate success [38, 39]. Another method is through genetic modulation, in particular transfection of stem cells with recombinant DNA encoding for proteins that regulate the commitment to the keratinocyte lineage [40]. Although this method presents with exciting new potential, one cannot overlook the potential detrimental effects and safety concerns of genetic manipulation of stem cells [41].

### 5.4. Growth Factors

Of the known growth factors with a proposed role in wound healing, therapeutic efficacy has been demonstrated only for becaplermin (recombinant human platelet-derived growth factor, Regranex) in several randomized controlled clinical trials [42]. Nevertheless, recent data reported an increased cancer risk in patients treated with more than three tubes of becaplermin so that pending lower follow-up data on the potential risk of malignancy in connection with its use this agent should be used with extreme caution in patients with diagnosed malignancy [43].

### 5.5. Subatmospheric Pressure Dressings

The use of subatmospheric pressure dressings such as the commercially available vacuum-assisted closure (VAC) device have been shown to be an effective way in accelerating the healing of various wounds. This technique optimizes blood flow, decreases local tissue edema, and removes excessive fluid from the wound bed. Additionally, the cyclical application of sub-atmospheric pressure alters the cytoskeleton of the cells in the wound bed thereby triggering a cascade of intercellular signals that increases the rate of cell division and formation of granulation tissue. The success rate of skin grafting is significantly increased when VAC is used as bolster covering the freshly skin-grafted wound [44, 45]. A recent review assessing current modalities in the treatment of diabetic foot ulcers [46] concluded that although vacuum compression therapy has been linked to significant reduction in wound area [47] and time to healing [48], this treatment was not shown to be costeffective and should therefore be used only in exceptional circumstances [49].

## 6. Perspectives and Conclusion

**Figure 1:**
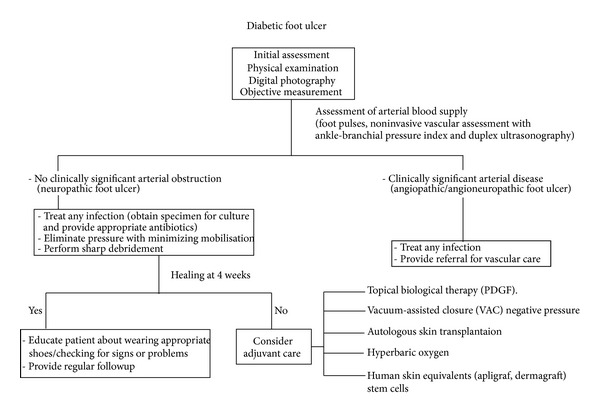
Algorithm for the management of diabetic foot ulcers.

The treatment of diabetic foot ulcers is a constant challenge in diabetes care and requires a multidisciplinary approach involving doctors, physiotherapists, specialised podologists, and orthopedic technicians. Over the recent years, novel and promising therapeutic options have emerged for the treatment of chronic diabetic foot ulcers, as summarized in Figure 1. However, clinical studies are needed in order to develop a well-structured algorithm for the assessment and treatment of diabetic ulcers to prevent lower-extremity amputations due to this complication.

## 7. Basic Conclusions

- The four phases of physiological wound healing are: haemostasis, inflammation, proliferation, and remodelling.
- Wound healing in diabetes is impaired by factors that are both extrinsic and intrinsic to the biology of wound.
- The standard treatment of diabetic ulcers includes optimization of glycemic control, extensive debridement, infection elimination, use of moisture dressings, and offloading high pressure.
- Current treatment methods in persistent diabetic foot ulcers include autologous skin transplantation, tissue-engineered human skin equivalents, bone marrow derived cells, growth factors, and subatmospheric pressure dressings.

## Abbreviations

AGEs: Advanced glycation end-products
ECM: Extracellular matrix
EPCs: Endothelial progenitor cells
HbA1c: Glycosylated haemoglobin
IDE: Insulin degrading enzyme
IL-1: Interleukin 1
IL-6: Interleukin 6
MMPs: Matrix metalloproteinases
PDGF: Platelet-derived growth factor
SDF-1*α*: Stromal cell-derived factor-1-alpha
TIMPs: Tissue inhibitors of metalloproteins
TNF-*α*: Tumour necrosis factor-*α*.
